# COVID‐19 vaccine acceptance among pregnant women and the reasons for hesitancy: A multi‐centre cross‐sectional survey

**DOI:** 10.1111/ajo.13622

**Published:** 2022-10-19

**Authors:** Monica Rikard‐Bell, James Elhindi, Justin Lam, Sean Seeho, Kirsten Black, Sarah Melov, Greg Jenkins, Justin McNab, Kerrie Wiley, Dharmintra Pasupathy

**Affiliations:** ^1^ Department of Obstetrics & Gynaecology Westmead Hospital Sydney New South Wales Australia; ^2^ Reproduction and Perinatal Centre, Faculty of Medicine and Health University of Sydney Sydney New South Wales Australia; ^3^ Department of Obstetrics & Gynaecology Royal North Shore Hospital Sydney New South Wales Australia; ^4^ Women and Babies Research, Kolling Institute, Faculty of Medicine and Health University of Sydney Sydney New South Wales Australia; ^5^ Central Clinical School, Royal Prince Alfred Hospital University of Sydney Sydney New South Wales Australia; ^6^ Westmead Institute of Maternal Fetal Medicine Westmead Hospital Sydney New South Wales Australia; ^7^ School of Health Sciences, Faculty of Medicine and Health University of Sydney Sydney New South Wales Australia; ^8^ Faculty of Medicine and Health, School of Public Health University of Sydney Sydney New South Wales Australia

**Keywords:** COVID‐19, COVID‐19 vaccines, mRNA vaccine, pregnancy, surveys and questionnaires

## Abstract

**Background:**

On 9 June 2021, the Australian Technical Advisory Group on Immunisation and Royal Australian and New Zealand College of Obstetricians and Gynaecologists recommended that pregnant women receive Comirnaty (Pfizer) messenger RNA vaccine at any stage of pregnancy.

**Aim:**

This multi‐centre study aimed to assess vaccine acceptance, reasons for hesitancy and determine if differences exist between health districts, to inform future policy strategies for COVID‐19 vaccination in pregnancy.

**Materials and methods:**

An online survey (developed based on the World Health Organization Behavioural and Social Drivers survey and modified for the pregnant population) was administered to a sample population of pregnant women attending antenatal clinics at two metropolitan hospitals (Westmead and Royal North Shore Hospital (RNSH)) in New South Wales between 15 September 2021 and 22 October 2021.

**Results:**

There were 287 pregnant women surveyed (Westmead 198 (69%), RNSH 66 (23%), no site 23 (8%)). There was a significantly lower Socio‐Economic Indexes for Areas score (5.66 vs 9.45, *P* = 0.001), fewer women born in Australia (37% vs 53%, *P* = 0.02) and higher number of children (0.77 vs 0.41, *P* = 0.01) among Westmead respondents. There was lower vaccination uptake (68% vs 86%, *P* = 0.01) and willingness to receive vaccine (68% vs 88% *P* = 0.01) at Westmead compared to RNSH. There was an increased proportion of respondents who were concerned that the vaccine could cause harm to the unborn baby at Westmead (38% vs 11%, *P* = 0.01).

**Conclusions:**

Along with healthcare provider recommendation for vaccination in pregnancy, materials should be targeted to specific safety concerns of pregnant women.

## INTRODUCTION

**Figure 1:**
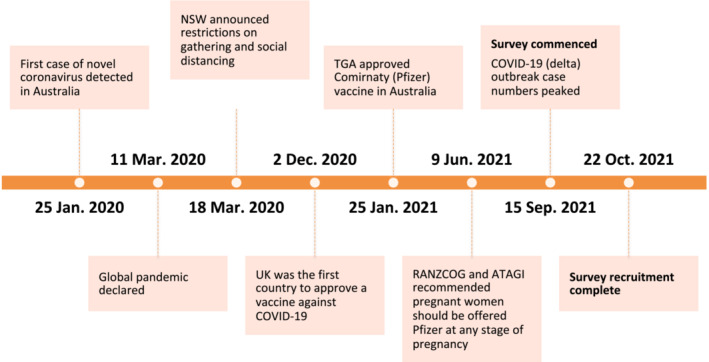
Timeline of events for COVID‐19 outbreak and vaccine recommendations in Australia. ATAGI, Australian Technical Advisory Group on Immunisation; NSW, New South Wales; RANZCOG, Royal Australian and New Zealand College of Obstetricians and Gynaecologists; TGA, Therapeutic Goods Administration.

The World Health Organization (WHO) declared COVID‐19 a global pandemic on 11 March 2020.^1^ The first case of novel coronavirus in Australia was reported on 25 January 2020. On 18 March 2020, New South Wales (NSW) announced the beginning of restrictions on gathering and social distancing.^2^ The first country to approve a vaccine against COVID‐19 was the United Kingdom on 2 December 2020. The Therapeutic Goods Administration (TGA) in Australia approved the use of Comirnaty (Pfizer) on 25 January 2021 for people aged 16 years and over.^3^ There was insufficient evidence to recommend the routine use of Comirnaty (Pfizer) vaccine in pregnancy until 9 June 2021, when the Royal Australian and New Zealand College of Obstetricians and Gynaecologists (RANZCOG) and the Australian Technical Advisory Group on Immunisation (ATAGI) advised that pregnant women should be offered the Comirnaty (Pfizer) messenger RNA (mRNA) vaccine at any stage of pregnancy (Fig. 1).^4^ This is because the risk of severe outcomes from COVID‐19 is significantly higher for pregnant women and their unborn babies.^5^ This recommendation changed to include pregnant women following the publication of a large study confirming the safety of mRNA COVID‐19 vaccines in pregnancy in the United States.^5^

Vaccine hesitancy is defined by the WHO as the reluctance or refusal to vaccinate despite the availability of vaccines.^6^ The WHO identified vaccine hesitancy as a major barrier which threatens to reverse progress made in tackling vaccine‐preventable diseases.^6^ Existing research regarding vaccination hesitancy in pregnancy is mostly surrounding influenza vaccination. Common barriers to influenza vaccine acceptance by pregnant women include concerns about the safety of the vaccine for their unborn child and fear that the vaccine has not been properly tested. Risk perception of the disease and the vaccine are a major factor in maternal vaccination behaviour during pregnancy.^7^ A multi‐centre international study surveying 16 countries and including 5294 pregnant women, found that COVID‐19 vaccine acceptance in this group was among the lowest in Australia.^8^ Therefore, it was hypothesised that vaccine hesitancy may be high and differ between obstetric populations in NSW; however there were no studies to confirm this and the potential reasons for hesitancy were unknown.

The aim of this multi‐centre study was to assess rates of COVID‐19 vaccine acceptance and reasons for vaccine hesitancy in different obstetric populations to inform development of policy and practice strategies for COVID‐19 vaccination in pregnancy.

## MATERIALS AND METHODS

### Study design

Pregnant women attending two antenatal clinics in different areas of Sydney, Australia, were invited to complete an online survey between 15 September 2021 and 22 October 2021.

### Survey development

The survey was developed by a multi‐disciplinary group of global experts based on the WHO Behavioural and Social Drivers (BeSD) Model of COVID‐19 vaccination behaviour,^9^ and psychometrically validated among adults in low‐middle income and high‐income countries.^10^ The BeSD Model of COVID‐19 vaccine behaviour has four domains: what people feel and think about vaccination; social processes; motivation; and practical factors. The survey was modified for relevance to the Australian obstetric population, with input from a group of specialists in obstetrics, epidemiology, and social sciences (authors 2, 3, 6, 7 and 8). Survey components included demographic and pregnancy data, and questions covering the four BeSD domains, modified to include additional questions relating to risk to the unborn baby (Appendix S1). The survey items were measured using a Likert scale or closed‐ended responses.

### Study population

Pregnant women, regardless of gestation, attending antenatal clinic waiting rooms and telehealth appointments in two tertiary metropolitan hospitals in Sydney, Australia, were invited to participate. Interested participants were directed to a QR code on posters and brochures which upon scanning directed them to an online survey; consent was implied by completing the survey. These two sites are recognised to have wide demographic and socioeconomic diversity. Westmead Hospital is located in the western suburbs of Sydney which facilitates approximately 5000 births annually with 42.2% of mothers born in English speaking countries. Royal North Shore Hospital (RNSH) is in the northern suburbs of Sydney which facilitates approximately 3000 births annually and 56.9% of mothers were born in English speaking countries.^11^

Recruitment was undertaken between 15 September and 22 October 2021. The recruitment period of the survey coincided with the outbreak of the delta variant of COVID‐19 in NSW.

### Sample size

We used a convenience sample of 300 women recruited over a period of four weeks. Recruitment was conducted from all clinics at both sites, ensuring participants were included from a range of care models from low‐risk midwifery to high‐risk obstetrics. The four‐week duration of the survey was chosen as a function of timeliness: due to the rapidly changing pandemic environment, understanding the attitudes, feelings and risk assessment of pregnant women was paramount to informing COVID‐19 vaccination policy and practice within the clinics.

### Data analysis

Data were analysed as an entire cohort, and then by hospital site. Surveys that had no site recorded were excluded from the analysis. Data were analysed using a *t*‐test with unequal variances assumed. Data samples were verified graphically to ensure that assumptions of underlying normal distribution apply. A significance threshold of *P* = 0.05 was applied.

Data were analysed using Stata SE 14.2 – StataCorp LLC.

### Human research ethics approval

Ethics approval was gained from Western Sydney Local Health District (WSLHD) Covid Executive Committee (Human Research Ethics Committee) and the WSLHD Research Governance Officer. 2021/PID02308‐2021/ETH11155.

## RESULTS

During the study period, 287 pregnant women were surveyed, comprising 198 (69%) at Westmead and 66 (23%) at RNSH, but 23 (8%) respondents did not specify a centre and were therefore excluded from the analyses. On average, respondents completed 85% of questions and spent 10–15 min on the survey.

Overall there was no significant difference in maternal age, marital status or level of education between the two sites (Table 1). However, there was a significant difference in Socio‐Economic Indexes for Areas (SEIFA) score, Australian birth, number of household members and previous children. This reflects the known demographic differences between the two populations. The sample population was found to approximate the NSW perinatal data for maternal age (NSW average 31.1 years). However, survey participants were less likely to be Australian born (41%), compared with NSW perinatal data (61.2%).^13^

**Table 1:** Demographics of pregnant women surveyed about COVID‐19 vaccination in two antenatal clinics in Sydney

Characteristic	Total responders	Entire cohort (*N* = 260)	Westmead (*n* = 196)	RNSH (*n* = 64)	Between‐groups *P*‐value*
Age, years	260	32.8 (SD 5.1)	32.6 (SD 5.1)	33.6 (SD 5.3)	0.16
SEIFA score, 0–10	257	6.6 (SD 3.0)	5.66 (SD 2.8)	9.45 (SD 1.0)	<0.001
Born in Australia, *n* (%)	259	106 (41%)	72 (37%)	34 (53%)	0.02
Years lived in Australia, no. of years	253	18.3	17.0	21.9	0.01
% of years of life in Australia†	253	55.9%	52.7%	65.3%	0.02
Language other than English at home	258	159 (62%)	127 (65%)	32 (50%)	0.03
Highest education level					
Completed primary school	253	2 (1%)	2 (1%)	0 (0%)	0.59
Completed secondary school		21 (8%)	17 (9%)	4 (6%)	
More than secondary school		228 (90%)	168 (89%)	60 (94%)	
No formal education		2 (1%)	2 (1%)	0 (0%)	
Marital status					
De facto	258	35 (13%)	22 (11%)	13 (20%)	0.13
Divorced		4 (2%)	2 (1%)	2 (3%)	
Married		199 (77%)	157 (81%)	42 (66%)	
Never married		16 (6%)	10 (5%)	6 (9%)	
Separated		4 (2%)	3 (2%)	1 (2%)	
Household members, *n*	257	2.97	3.07	2.68	0.03
Previous children, *n*	221	0.68	0.77	0.41	0.01

RNSH, Royal North Shore Hospital; SEIFA, Socio Economic Index for Areas.^12^

† The percentage the participant's life that they have spent in Australia.

* Two‐sample *t*‐test.

There was no significant difference in gestational age between the two cohorts with a mean gestational age of 29 weeks (Table 2). There was no significant difference in participants having previously received (any) vaccination as an adult (87%) or (any) vaccination in pregnancy (73%) between the sites. One hundred and seven (73%) participants had already received the COVID‐19 vaccination at the time of survey completion with lower rates of COVID‐19 vaccination observed in respondents at Westmead compared to RNSH (68% vs 86%; *P* = 0.01). The motivation questions were also analysed by isolating just those women who had not received the Covid‐19 vaccine (27%). Sixty‐one percent of women who had not received the COVID‐19 vaccine said they would ‘not’ get it in pregnancy, 30% ‘unsure’ and 9% responded ‘yes’. When asked how much they wanted to receive the COVID‐19 vaccine while pregnant, 65% responded ‘not at all’, 21% ‘a little’, 9% ‘moderately’ and 5% ‘very much’.

**Table 2:** COVID‐19 vaccination survey results grouped by category

Characteristic	Total responders	Entire cohort	Westmead	Royal North Shore Hospital	*P*‐value
Medical/vaccination history of survey participants					
Gestational age?	256	29.1	29.2	28.7	0.65
Previous vaccination (any) as an adult?	253	236 (93%)	173 (92%)	63 (98%)	0.08
Previous vaccination (any) in pregnancy?	253	219 (87%)	160 (85%)	59 (92%)	0.14
Have you been infected with COVID‐19?	259	11 (4%)	9 (5%)	2 (3%)	0.59
Have you already received the COVID‐19 vaccination?	212	155 (73%)	107 (68%)	48 (86%)	0.01
Social and behavioural data: motivation to obtain COVID‐19 vaccination					
If a COVID‐19 vaccine is available to you will you get it in pregnancy?					
Yes	232	170 (74%)	116 (68%)	54 (88%)	0.01
No		40 (17%)	36 (21%)	4 (7%)	
Not Sure		22 (9%)	19 (11%)	3 (5%)	
How much do you want to get the COVID‐19 vaccine while you are pregnant?					
Very much	220	108 (49%)	76 (47%)	32 (56%)	0.02
Moderately		44 (20%)	28 (17%)	16 (28%)	
A little		20 (9%)	15 (9%)	5 (8%)	
Not at all		48 (22%)	43 (27%)	5 (8%)	
Thinking and feeling					
How concerned are you about getting COVID‐19?					
Very concerned	254	80 (32%)	63 (33%)	17 (26%)	0.54
Moderately		80 (32%)	59 (31%)	21 (32%)	
A little concerned		78 (30%)	54 (29%)	24 (37%)	
Not at all		16 (6%)	13 (7%)	3 (5%)	
How much would you trust the new COVID‐19 vaccine?					
Very much	232	100 (43%)	71 (42%)	29 (48%)	0.34
Moderately		82 (35%)	58 (34%)	24 (39%)	
A little		23 (10%)	19 (11%)	4 (6.5%)	
Not at all		27 (12%)	23 (13%)	4 (6.5%)	
How important is getting a COVID‐19 vaccine in pregnancy for your health?					
Very important	232	153 (66%)	106 (62%)	47 (77%)	0.23
Moderately important		40 (17%)	32 (19%)	8 (13%)	
A little important		20 (9%)	17 (10)	3 (5%)	
Not at all important		19 (8%)	16 (9%)	3 (5%)	
How important is getting a COVID‐19 vaccine in pregnancy for your baby's health?					
Very important	232	138 (59%)	100 (58%)	38 (62%)	0.71
Moderately important		50 (22%)	37 (22%)	13 (21%)	
A little important		24 (10%)	17 (10%)	7 (12%)	
Not at all important		20 (9%)	17 (10%)	3 (5%)	
How much do you think a COVID‐19 vaccine for yourself will protect others in your community?					
Very much	232	148 (64%)	109 (64%)	39 (64%)	0.3
Moderately		49 (21%)	33 (19%)	16 (26%)	
A little		21 (9%)	16 (9%)	5 (8%)	
Not at all		14 (6%)	13 (8%)	1 (2%)	
How safe do you think a COVID‐19 vaccine will be for you in pregnancy?					
Very safe	232	92 (39%)	72 (42%)	20 (33%)	0.07
Moderately safe		85 (37%)	55 (32%)	30 (49%)	
A little safe		25 (11%)	18 (11%)	7 (11%)	
Not at all safe		30 (13%)	26 (15%)	4 (7%)	
How concerned are you that a COVID‐19 vaccine could cause you to have a serious reaction?					
Very concerned	232	59 (26%)	52 (30%)	7 (11%)	0.01
Moderately		54 (23%)	42 (25%)	12 (20%)	
A little concerned		81 (35%)	53 (31%)	28 (46%)	
Not at all		38 (16%)	24 (14%)	14 (23%)	
How concerned are you that having a COVID‐19 vaccine while pregnant could cause harm to your unborn baby?					
Very concerned	231	72 (31%)	65 (38%)	7 (11%)	0.01
Moderately		51 (22%)	34 (20%)	17 (28%)	
A little concerned		70 (30%)	44 (26%)	26 (43%)	
Not at all		38 (17%)	27 (16%)	11 (18%)	
Social processes influencing uptake of COVID‐19 vaccination					
In your family, who will have the final say about getting a COVID‐19 vaccine?					
Me	216	176 (81%)	127 (80%)	49 (86%)	0.21
Not only me		40 (19%)	32 (20%)	8 (14%)	
Do you think your close family and friends would want you to get a COVID‐19 vaccine?					
Yes	216	157 (73%)	110 (69%)	47 (82%)	0.19
No		20 (9%)	17 (11%)	3 (5%)	
Not sure		39 (18%)	32 (20%)	7 (13%)	
Do you think your community leaders would want you to get a COVID‐19 vaccine?					
Yes	216	180 (83%)	126 (79%)	54 (95%)	0.302
No		4 (2%)	4 (3%)	0 (0%)	
Not sure		32 (15%)	29 (18%)	3 (5%)	
Do you think your religious leaders would want you to get a COVID‐19 vaccine?					
Yes	215	111 (52%)	79 (50%)	32 (56%)	0.42
No		11 (5%)	10 (6%)	1 (2%)	
Not sure		93 (43%)	69 (44%)	24 (42%)	
Do you think most adults you know will get a COVID‐19 vaccine, if it is recommended to them?					
Yes	216	183 (85%)	130 (82%)	53 (93%)	0.13
No		7 (3%)	6 (4%)	1 (2%)	
Not sure		26 (12%)	23 (14%)	3 (5%)	
Do you think getting a COVID‐19 vaccine will allow you to safely see your family and friends again?					
Very much	215	132 (62%)	100 (63%)	32 (56%)	0.08
Moderately		50 (23%)	30 (19%)	20 (35%)	
A little		20 (9%)	17 (11%)	3 (5%)	
Not at all		13 (6%)	11 (7%)	2 (4%)	
How much do you trust the (healthcare providers) who would give you a COVID‐19 vaccine?					
Very much	216	119 (55%)	86 (54%)	33 (58%)	0.12
Moderately		66 (31%)	47 (30%)	19 (33%)	
A little		18 (8%)	13 (8%)	5 (9%)	
Not at all		13 (6%)	13 (8%)	0	
Have you seen or heard anything bad about COVID‐19 vaccines?					
Yes	213	180 (85%)	128 (82%)	52 (93%)	0.05
No		33 (15%)	29 (18%)	4 (7%)	
Practical issues in obtaining the COVID‐19 vaccination					
Do you know where to go to get a COVID‐19 vaccine?					
Yes	232	230 (99%)	171 (100%)	59 (97%)	0.07
No		2 (1%)	0	2	
How easy is it to get a COVID‐19 vaccine?					
Very easy	232	103 (45%)	83 (49%)	20 (33%)	0.03
Moderately easy		75 (32%)	55 (32%)	20 (33%)	
A little easy		37 (16%)	25 (15%)	12 (20%)	
Not easy		17 (7%)	8 (4%)	9 (14%)	
What makes it hard?					
Nothing, it's not too hard	229	147 (64%)	119 (70%)	28 (47%)	0.01
Wait time too long		49 (21%)	32 (19%)	17 (28%)	
Booking system difficult		29 (13%)	15 (9%)	14 (23%)	
I cannot go on my own		4 (2%)	3 (2%)	1 (2%)	
Where would you prefer to get a COVID‐19 vaccine?					
General practice	220	68 (31%)	44 (27%)	19 (33%)	0.48
COVID‐19 vaccination hub		52 (24%)	28 (17%)	6 (10%)	
Antenatal clinic		70 (32%)	31 (19%)	14 (24%)	
Other		30 (13%)	59 (37%)	19 (33%)	

The level of concern about getting COVID‐19 disease was consistent across both sites. Trust in the vaccine and importance of the vaccine for themselves, their baby or others was consistent across both sites.

Questions on the perceived safety of the vaccine revealed that only 39% of respondents thought COVID‐19 vaccine is ‘very safe’, with 26% ‘very concerned’ they could have a serious reaction. Thirty‐one percent of respondents reported being ‘very concerned’ that having a COVID‐19 vaccine while pregnant could cause harm to their unborn baby; this was significantly higher in the Westmead population compared to RNSH.

Eighty‐one percent of respondents reported that in their family they would have the final say about getting a COVID‐19 vaccine with similar rates among respondents from both sites (Table 2). There was a high level of trust in healthcare providers across both sites with 86% of respondents reporting they ‘very much’ or ‘moderately’ trust healthcare providers.

Almost all participants (99%) knew where to go to get a COVID‐19 vaccine and the preferred place to get the vaccine was equally split between general practitioner, vaccination hub and antenatal clinic.

## DISCUSSION

The majority of pregnant women in this study had received a COVID‐19 vaccine; however, significantly fewer from Westmead took up the vaccine, compared with RNSH. This finding does not correlate with the general population vaccination rates by region, which showed that the Westmead area had higher vaccination rates than the RNSH area.^18^ Similarly, fewer women from Westmead said they wanted the vaccine while pregnant, compared with RNSH. Of those who had not received a vaccine, most indicated they would not receive one while pregnant or were not sure, indicating that most of the unvaccinated women in this sample had a level of hesitancy. Below we discuss our findings in relation to the demographics of the sample and the different domains of the BeSD Model.

### Demographics

Demographically, the respondents were largely similar between both sites, except that respondents from Westmead were generally more socioeconomically disadvantaged, had more previous children, and were less likely to be born in Australia. Previous studies have identified belonging to an ethnic minority or being multiparous are associated with lower vaccine uptake in pregnancy, although this appears to be variable for different vaccines.^24^ The population surveyed were highly educated with more than 90% of participants having higher than secondary education, compared with the background rate for completing high school in the Westmead area (68.2%) and the RNSH area (78.9%).^11^ Low health literacy and education level have been associated with reluctance to accept a COVID‐19 vaccine,^14^ thus it is possible that the vaccine uptake among this sample may be an overestimation of uptake in the broader antenatal population in these areas.

**Figure 2:**
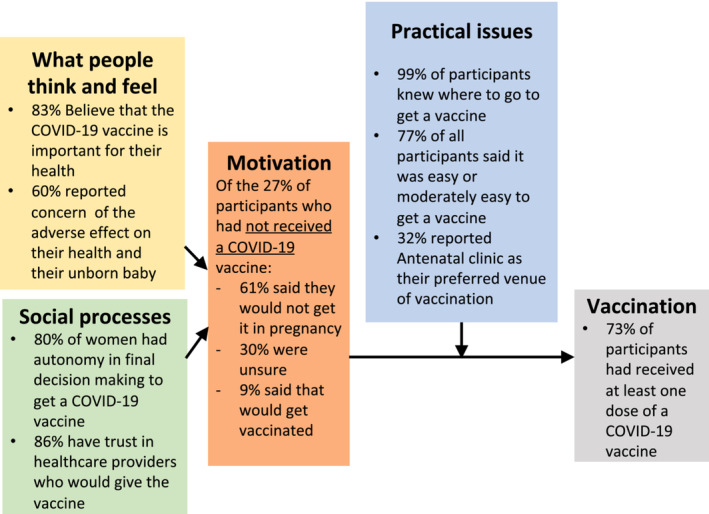
COVID‐19 vaccination survey results by domain.

Lower household income and living in disadvantaged areas has previously been associated with increased likelihood of COVID‐19 vaccine hesitancy^15^ which may to some extent explain the comparatively lower levels of vaccination in the Westmead sample. Similarly, 65% of women from the Westmead sample spoke a language other than English at home, compared with 50% from the RNSH sample. Previous studies suggest that people who speak a language other than English at home experience more difficulty in finding information and understanding government messaging around COVID‐19.^16^ Research with Australian culturally and linguistically diverse (CALD) communities suggests that partnerships between CALD communities and government are paramount for effective health communication, and that changing behaviour requires tailoring solutions for community needs that move beyond simply disseminating information (Fig. 2).^17^

### Thinking and feeling

Up to 60% of women were concerned about the adverse effect of vaccine on their health and that of their unborn baby, with rates higher in Westmead compared to RNSH, which may explain some of the hesitancy seen among those who were unvaccinated. However, two‐thirds agreed that getting a COVID‐19 vaccine was very important for their health. This demonstrates that women generally valued COVID‐19 vaccination, but were understandably concerned for their baby's health and the safety of the vaccine. Providing reassurance to pregnant women regarding the safety of vaccination to both mother and fetus while focusing on the benefits, will likely help alleviate hesitancy borne of safety concerns. Engagement of women in the antenatal clinic and motivational interviewing techniques may help support vaccination decisions.^19^

### Social processes

More than 80% of women felt they had the final say on whether to get the COVID‐19 vaccine, and the majority felt those around them would want them to be vaccinated. In the context of the significant cultural diversity in the population serviced by these maternity units, this finding informed the decision to target the local vaccination program at pregnant women themselves rather than their spouses or other decision makers within the family. That most women felt those around them wanted them to be vaccinated suggests that any hesitancy was unlikely related to social and cultural norms of this sample.

We observed a high level of trust in healthcare providers across both sites and this has previously been positively correlated with intent to vaccinate in the pregnant population.^7^, ^22^ Provider recommendation is known to be strongly associated with uptake of COVID‐19 vaccine and other recommended pregnancy vaccines.^19^, ^20^, ^23^ Providers have reported challenges staying across the large volume of COVID‐19 vaccine safety information. Developing clear, simple materials for antenatal care providers to support conversations with their patients would support confident recommendations.^21^

### Practical issues

Vaccine uptake does not seem to be affected by access to vaccination in this sample. Almost all participants knew where to go to get a COVID‐19 vaccine, suggesting that public health messaging and media were prominent and effective at that stage of the pandemic response. Additionally, a large proportion of women reported it was very or moderately easy to get a COVID‐19 vaccine and this was higher in the Westmead area, with note that at the time COVID‐19 vaccination was not available at either antenatal clinic and women had to source the vaccine elsewhere. However, one‐third of women reported their preferred location to receive a COVID‐19 vaccine was in an antenatal clinic. Previous studies demonstrated that point of care vaccination for pregnant women increased awareness and acceptance of vaccination;^22^ this may be an avenue to improve ease and access for pregnant patients.

### Summary

This study contributes important knowledge to the drivers of COVID‐19 vaccination in pregnancy, and is one of the first that we are aware to apply the BeSD framework and survey to an obstetric population. Limitations of the study include the small numbers at RNSH and that the survey was not translated into different languages due to time and cost restraints, therefore potentially missed a proportion of women who cannot speak English, where health literacy may be poorer. This is particularly relevant in a culturally diverse population as the sample may be biased toward women with higher education and increased health literacy.

Vaccination rates in the pregnant population are lower than the general population and hesitancy is important to address for the health of women and their babies. Our findings demonstrate that trust and confidence in vaccine safety appear to be significant drivers of the difference in vaccination rates observed in both sites. We have demonstrated that level of concern varies between populations, therefore strategies to optimise vaccination rates will be most effective if they are tailored to be site‐specific.

Worldwide data have confirmed the need for booster campaigns and a multi‐faceted and locally specific approach is needed to increase vaccination uptake with an emphasis on safety and benefits for the fetus. Pregnant women are frequently seen by healthcare providers during pregnancy, which provides opportunity to educate, engage and motivate women regarding vaccination. Resources to support providers in having such conversations with pregnant women about COVID‐19 vaccination are also needed.

## Supporting information


**Appendix S1.** Survey.
