# Supplementary material for: COVID‐19 vaccine acceptance among pregnant women and the reasons for hesitancy: A multi‐centre cross‐sectional survey
Source: Aust N Z J Obstet Gynaecol. 2022 Oct 19:10.1111/ajo.13622. Online ahead of print. doi: 10.1111/ajo.13622 (PMC9874455; doi:10.1111/ajo.13622)
Supplement: Supplementary file 1 — Appendix S1. Survey. [file AJO-9999-0-s001.docx]

APPENDIX S1: survey

DEMOGRAPHICS

| 1 |  | |  | Age |
| --- | --- | --- | --- | --- |
| 2 |  | |  | Postcode of residence |
| 3 |  | |  | What country were you born in? |
| 4 |  | |  | Years lived in Australia |
| 5 | |  |  | What language do you mainly speak at home? |
| 6 | |  | Education level | What is the highest level of formal education you have received?  - No formal education - Did not complete primary (less than 6 years) - Completed primary school - Completed secondary school - More than secondary school  What is the total number of years of formal schooling that you have received? |
| 7 | |  | Marital status | Married  De facto  Separated  Single |
| 8 | |  | How many family members / individuals share your house (including yourself) |  |

PREGNANCY / MEDICAL

| 9 |  | How many weeks pregnant are you? |  |
| --- | --- | --- | --- |
| 10 |  | Do you have any other children? | No  Yes  If yes – how many? |
| 11 |  | Have you been previously diagnosed with COVID-19 | A. To your knowledge, are you or have you been infected with COVID-19?   - YES - NO   B. IF YES   - did you require hospitalisation   Was it confirmed by a test?   - confirmed by a test - not confirmed by a test |
| 12 | | Perceived risk – self | How concerned are you about getting COVID-19 disease?   - not at all concerned - a little concerned - moderately concerned - very concerned |
| 13 | | General vaccination | A. Have you ever received a vaccine as an adult?  YES / NO  B. Have you ever received a vaccine in pregnancy?  YES / NO  C. Have you received the COVID-19 vaccination in pregnancy?  YES / NO |

COVID-19 VACCINATION

| 14 |  | Do you know where to go to get a COVID-19 vaccine?  YES/NO |
| --- | --- | --- |
| 15 |  | How easy is it to get a COVID vaccine for yourself? would you say   - not at all easy - a little easy - moderately easy - very easy |
| 16 |  | What makes it hard for you to get a COVID vaccine?   - nothing. Its not hard - vaccination costs too much - the booking system is difficult to use - the appointment wait time is too long - I can’t go on my own (I have a physical limitation) - The vaccination site is too far away - The opening times are inconvenient |
| 17 | COVID-19 vaccine – trust in a new vaccine | How much would you trust the new COVID-19 (Pfizer) vaccine if it were available for you now?   - not at all - a little - moderately - very much - Please explain the reason (s) why you trust or do not trust the COVID-19 vaccine (free text) |
| 18 | COVID-19 vaccine – confidence in benefits | A. How important do you think getting a COVID-19 vaccine in pregnancy will be for your health? Would you say…   - not at all important - a little important - moderately important - very important   B. How important do you think getting a COVID-19 vaccine in pregnancy will be for your baby’s health?   - Not at all important - a little important - moderately important - very important |
| 19 | COVID-19 vaccine – confidence in protecting others | How much do you think getting a COVID-19 vaccine for yourself will protect other people in your community for COVID-19?   - not at all - a little - moderately - very much |
| 20 | COVID-19 vaccine – confidence in vaccine safety (safe) | How safe do you think a COVID-19 vaccine will be for you in pregnancy? Would you say…   - not at all safe - a little safe - moderately safe - very safe |
| 21 | COVID-19 vaccine – confidence in vaccine safety (harms) | A. How concerned are you that a COVID-19 vaccine could cause you to have a serious reaction? Would you say   - not at all concerned - a little concerned - moderately concerned - very concerned   B. How concerned are you that having a COVID-19 vaccine while you’re pregnant could cause harm to your unborn baby? Would you say   - not at all concerned - a little concerned - moderately concerned - very concerned |
| 22 | COVID-19 vaccine - intention | If a COVID-19 vaccine is available to you will you get it, in pregnancy?   - Yes - No - Not sure   If answered no/not sure   - Would you get a COVID-19 vaccine when you’ve had the baby? - yes - no - not sure |
| 23 | COVID-19 vaccine – extent of intention | How much do you want to get a COVID-19 vaccine while you’re pregnant? Would you say….   - not at all - a little - Moderately - very much |
| 24 | COVID-19 vaccine – preferred site for vaccination | Where would you prefer to get a COVID-19 vaccine?   - COVID-19 vaccine centre/hub - hospital / Antenatal clinic - health centre/clinic - GP clinic - workplace - pharmacy - community centre/ hall - somewhere else, specify - I don’t want the vaccine   Please give your reason for preferring this location/service?   - Free text |
| 25 | COVID-19 vaccine – decision autonomy | In your family, who will have the final say about whether you get a COVID-19 vaccine?   - me - my spouse/partner - my mother - my father - my mother-in-law - my father-in-law - my daughter - my son - someone else, please specify |
| 26 | COVID-19 vaccine – family norms | Do you think most of your close family and friends would want you to get a COVID-19 vaccine?   - yes - no - not sure |
| 27 | COVID-19 vaccine – community and religious leader norms | Do you think your community leaders would want you to get a COVID-19 vaccine?   - yes - no - not sure - Do you think your religious leaders would want you to get a COVID-19? - yes - no - not sure |
| 28 | COVID-19 vaccine – descriptive social norms | Do you think most adults you know will get a COVID-19 vaccine, if it is recommended to them?   - yes - no - not sure |
| 29 | COVID-19 vaccine – safe to see friends | Do you think that getting a COVID-19 vaccine will allow you to safely see your family and friends again? (tick box)   - not at all - a little - moderately - very much |
| 30 | COVID-19 vaccine – confidence in providers | How much do you trust the (healthcare providers) who would give you a COVID-19 vaccine? Would you say you trust them?   - not at all - a little - moderately - very much - Please explain the reason (s) for your answer - Free text |
| 31 | COVID-19 vaccine – negative information | Have you seen or heard anything bad about COVID-19 vaccines?   - yes - no - What did you see/hear? - Free text |

32.

How do you feel about COVID-19 vaccines? (FREE TEXT)

Please provide your contact details if you would like to be contacted a more detailed interview.

First name / Last name

Phone number
